# Gastric Emptying and Dynamic In Vitro Digestion of Drinkable Yogurts: Effect of Viscosity and Composition

**DOI:** 10.3390/nu10091308

**Published:** 2018-09-14

**Authors:** Olivia Ménard, Marie-Hélène Famelart, Amélie Deglaire, Yann Le Gouar, Sylvie Guérin, Charles-Henri Malbert, Didier Dupont

**Affiliations:** 1 Institut National de la Recherche Agronomique (INRA)-Agrocampus Ouest, Science et Technologie du Lait et de l’œuf (STLO), 65 rue de Saint-Brieuc, 35042 Rennes CEDEX, France; olivia.menard@inra.fr (O.M.); marie-helene.famelart@inra.fr (M.-H.F.); amelie.deglaire@agrocampus-ouest.fr (A.D.); yann.le-gouar@inra.fr (Y.L.G.); 2 Institut Nutrition-Métabolisme-Cancer, INRA, Institut National de la Santé et de la Recherche Médicale (INSERM), Université Rennes 1, Domaine de la prise, 35590 Saint-Gilles, France; sylvie.guerin@inra.fr; 3 INRA, ANI-SCAN Unit, Domaine de la Prise, 35590 Saint-Gilles, France; charles-henri.malbert@inra.fr

**Keywords:** gastric emptying, gamma-scintigraphy, yogurt, in vitro digestion, casein, whey protein, satiety

## Abstract

Gastric emptying of food is mainly driven by the caloric concentration, the rheological properties of the chyme, and the physical state (liquid/solid) of food once in the stomach. The present work investigated: (1) The effect of the composition and the viscosity of drinkable yogurts on gastric emptying in pigs, and (2) the behavior of yogurts during dynamic in vitro digestion. Three isocaloric liquid yogurts were manufactured: Two enriched in protein and fiber showing either a low (LV) or high (HV) viscosity, one control enriched in sugar and starch (CT). They were labelled with ^99m^Tc-sulfur colloid and given to pigs (*n* = 11) to determine gastric emptying pattern by gamma scintigraphy. Then dynamic in vitro digestion of the yogurts was done using the parameters of gastric emptying determined in vivo. Gastric emptying half-times were significantly longer for LV than CT, whereas HV exhibited an intermediate behavior. In vitro gastric digestion showed a quick hydrolysis of caseins, whereas whey proteins were more resistant in the stomach particularly for LV and HV. During the intestinal phase, both whey proteins and caseins were almost fully hydrolyzed. Viscosity was shown to affect the behavior of yogurt in the small intestine.

## 1. Introduction

Until recently, it was considered that the structure of the food matrix had a limited impact on food digestion and food could be described only according to its composition in proteins, lipids, and carbohydrates. During the last years, new evidences have clearly demonstrated that the food matrix structure plays a key role on the kinetics of transit and hydrolysis of the macronutrients [1,2,3,4]. Several parameters have been shown to affect food intake, transit, and digestion. Even before food arrives in the gastrointestinal tract, cognitive and sensory signals generated by the sight and smell of food and by the oro-sensory experience of food in the oral cavity influence the amount of food ingested for that eating episode, but also for an extended period of time during which no ingestion takes place. Aside the sensory component, the composition of food could also play a key role and the effect of the type of macronutrients on food intake has been further investigated in many studies. For example, dietary protein has been observed to increase satiety and suppress short-term food intake beyond what would be expected by an iso-energetic amount from carbohydrates and fats [5,6]. The development of protein-supplemented foods has been used as a strategy for either modulating appetite in healthy adults [7] or increasing the protein intake of elderly people suffering from malnutrition [8,9]. However, the extent to which the source of the protein matters is uncertain. Whey and casein, two milk derived protein sources, have more commonly been studied with regard to their effects on satiety and food intake. Whey proteins account for 20% of the total milk protein and are rich in essential amino acids, whereas casein is the major protein of milk accounting for ~80% of the total protein [10]. Whey protein and casein are both heterogeneous groups of proteins containing all amino acids and are especially rich in the essential ones, although in different proportions. Whey protein is reported as more satiating than casein [11,12], although this statement is still controversial. Indeed, a recent review indicates that whey is more satiating in the short term, whereas casein is more satiating in the long term [13] because of different mechanisms of action. Whey tends to stimulate the secretion of the incretin hormones glucagon-like peptide-1 (GLP-1) and glucose-dependent insulinotropic polypeptide, whereas casein is more active on the satiety (cholecystokinin, peptide YY) and hunger-stimulating (ghrelin) hormones.

Another food ingredient that can have beneficial effects on food intake is dietary fiber [14]. Fiber is thought to affect satiety in many ways, depending on the fiber type, and relating to its ability to bulk foods, increase viscosity, gel in the stomach, and ferment in the distal part of the gut [15]. When reviewing the literature available on the effect of fiber on satiety, it has been shown that fibers characterized as being more viscous (e.g., pectins, β-glucans and guar gum) reduced appetite more often than the less viscous fibers. However, overall, effects on energy intake and body weight were relatively small [16].

Post-ingestive signals encoding for nutrient content [17,18] and volume [19] arising from the stomach and intestine also affect satiety. Gastric signals are only of physical nature and are transmitted locally and centrally after the selective involvement of stretch receptors. These intramural receptors are sensitive to the amount of food present within the stomach and therefore are detecting the level of distension and the rate of emptying. Indirectly, via a chemical mediated duodenal detection of the amount of nutrient, and more specifically, the energy contents of the duodenal juice, where the satiation signals are transmitted centrally. Furthermore, part of the duodenal signal is transmitted to the enteric nervous system to alter locally gastric motility and emptying [20]. The increase in satiety through gastric distension has been demonstrated through alteration of the gastric emptying rate using isovolumetric and isocaloric liquid and semisolid meals [21,22,23].

Viscosity has been shown to have an effect on satiation and satiety in multiple studies [24,25,26,27] and it has been hypothesized that this effect of viscosity was due to its action on gastric emptying. Indeed, most of the published studies have shown that increasing the viscosity delayed the gastric emptying rate [23,27,28]. However, increasing the viscosity of the meal to slow down gastric emptying appears to be less effective than increasing its caloric content [29].

In this context, two fiber and protein-enriched yogurts (similar composition but different viscosity) and a control yogurt were formulated. All three yogurts had similar caloric content. The aim of the present study was to investigate whether protein and fiber enrichment could affect gastric emptying and, consequently, the kinetics of protein digestion. Yogurt digestion was studied using a dynamic in vitro model that had been previously validated against in vivo data [30]. In order to define the parameters of the model, gastric emptying half-time, and the shape of the emptying curve were determined by gamma-scintigraphy using the pig as a model. Gamma-scintigraphy, like Magnetic Resonance Imaging, is a direct method for assessing gastric emptying that has been shown to be more relevant than indirect methods such as ^13^C breath tests that can be biased by the food matrix as recently described [31]. Then, an in vitro dynamic digestion was conducted on the three yogurts using the parameters determined in vivo. The gastric and intestinal behavior of the three matrices were compared and the proteolysis kinetics were monitored.

## 2. Materials and Methods

Three isocaloric yogurts with different compositions and structure kindly provided by Senoble (Jouy, France) were submitted to in vivo and in vitro assays. The three yogurts, for which the composition is given in Table 1, had a control yogurt (CT) and two yogurts enriched in protein and fibers both with different textures, i.e., Low Viscosity (LV) or High Viscosity (HV).

### 2.1. Viscosity Analysis

Yogurt viscosity was measured using a cone-plan (6 cm diameter, 4 degree angle) geometry on an AR 2000 rheometer (TA Instruments, Leatherhead, UK). Yogurt was carefully sampled from the center of the cup, deposited on the plate at 4 °C and the cone was slowly lowered into the sample. A logarithm shear rate increase ranging from 0.2 to 100 s^−1^ at 4 °C was applied (*n* = 2) on 2 independent batches of the 3 yogurts. Since the study of gastric emptying on pigs and the in vitro digestion experiments occurred up to 15 days after manufacture, viscosity- of yogurts was assessed 4 days (D + 4) and -5 days (D + 15) after manufacture to check for a possible evolution of the viscosity during storage. Typical flow behaviors following the model of Herschel-Bulkley [32] were observed for yogurts. The apparent viscosities (Viscosity_app_) at 100 s^−1^ were deduced. They were significantly different for the 3 yogurts, but not between the days (Table 2).

### 2.2. Gastric Emptying Assessment by Gamma-Scintigraphy on Pigs

All procedures were in accordance with the European Community guidelines for the use of laboratory animals (L358-86/609/EEC). The study received prior approval by the local animal ethic committee (agreement number: R-2013-CHM-01). The facilities have the authorization to use animals (agreement number: A35-622) and radioisotopes (agreement number: T35-0282).

The animals used were 11 young Large White sows of about 3 months old and 30–35 kg body weight. During the week before the experiment, the animals were trained to consume the food while in quadrupedal position within a Pavlov stand. Furthermore, they were trained to eat 529 g (450 kcal) of yogurt within 5 min and then stay still for two hours in front of the gamma camera. On the day of experiment, conscious pigs were installed in a Pavlov stand and fed with 529 g of one of the three yogurt radiolabeled with 20 MBq, ^99m^Tc-colloid sulfur CK1 (CISBio International, Saclay, France), as described in previous studies [33]. The gamma camera was calibrated for energy and uniformity weekly. Data acquisition was performed using high-resolution low energy collimator and with a 64 × 64 pixel matrix. Gastric emptying was followed by gamma scintigraphy during 2 h after meal ingestion using sequential images taken every minute for 15 s each. During the 2 h of the experiment, animals had no access to water. Each animal received the 3 yogurts on separate days and each animal was therefore its own control.

The dynamic image series were analyzed with the OSIRIX MD software (Pixmeo SARL, Bernex, Switzerland) and with dedicated software. This software allowed to re-aligns the images to compensate the movements of the animal relative to the camera head during acquisition. Gastric emptying half-time (T_1/2_, time needed for 50% of the radioactivity to be transferred from the stomach into the small intestine) and the shape of the gastric emptying curve (β) that describes the length of the initial phase and the shape of the curve after the lag phase [34] were determined. The adequacy of the power exponential fit was evaluated using modified-stretch exponential (MSE).

### 2.3. Yogurt Dynamic In Vitro Digestion

A dynamic in vitro gastro-intestinal digestion system (DIDGI^®^, Institut national de la recherche agronomique (INRA), Rennes CEDEX, France) was used to simulate the digestion of the three yogurts and investigate the kinetics of milk protein hydrolysis during digestion. The gastric emptying half-time and the shape of the gastric emptying curve determined in vivo by gamma-scintigraphy on the three different matrices were entered in the simulator software to simulate the gastric emptying using the Elashoff equation [34]. Each matrix was digested in duplicate. In the gastric phase, aliquots were sampled at G0 (undigested yogurts) and after 120 min digestion (G120). In the intestinal phase, samples were taken after 180 min (I180) for the three yogurts and 240 min (I240) digestion for only the low and high viscosity yogurts (milk protein digestion in the control yogurt was already completed after 180 min). Except the gastric emptying parameters that were deduced from the in vivo experiment, the other parameters used in the digestion experiments were set up to simulate the adult gastro-intestinal, as proposed in Egger et al. [35]. They are summarized in Table 3.

### 2.4. SDS-PAGE

Milk protein extent of hydrolysis was assessed during the gastric and the intestinal phase by Sodium dodecyl sulfate polyacrylamide gel electrophoresis (SDS-PAGE) analysis. Briefly, the electrophoretic analyses were performed using 4–12% polyacrylamide NuPAGE^®^ Novex^®^ Bis-Tris 15 well precast gels (Invitrogen, Carlsbad, CA, USA) in accordance with the manufacturer’s instructions. All samples were diluted with NuPAGE^®^ LDS sample buffer and then treated with 0.5 M DL-dithiothreitol and distilled water. Mark 12 Unstained Standard (Invitrogen) was used as a molecular weight (MW) marker—as a reference of the position of the bands. Gels were fixed in 30% (*v/v*) ethanol, 10% (*v/v*) acetic acid, and 60% (*v/v*) deionized water. They were rinsed in deionized water before staining with Bio-Safe Coomassie stain (Bio-Rad Laboratories, Marnes-la-Coquette, France). Discoloration of gels were performed with water Image analysis of SDS-PAGE gels was carried out using Image Scanner III (General Electric (GE) Healthcare Europe GbmH, Velizy-Villacoublay, France). After digitization of gels, the bands were selected and their gray intensity determined by densitometry using the software Image Quant TL™ (GE Healthcare Europe GbmH, Velizy-Villacoublay, France). Densitometry analyses of the SDS-PAGE gels were used for the semi-quantification of protein levels. The percentage of each intact protein remaining in the gastric and intestinal compartment was estimated in comparison with the undigested yogurt sample.

### 2.5. Statistics

The difference of proteolysis kinetics between yogurts was examined by a two-way Analysis of variance (ANOVA) with yogurt and time as factors and with time as a repeated measure using the SAS software 9.3 (SAS, Cary, NC, USA). Post-hoc comparison was performed using a Tukey test. The value of *p* < 0.05 was considered statistically significant.

## 3. Results

### 3.1. Gastric Emptying

The results are summarized in Table 4 and an example of a video recorded is available as Supplementary Material. The T_1/2_ obtained for the LV yogurt was significantly different from the CT yogurt (*p* < 0.05) with 72.7 ± 5.1 and 57.7 ± 3.9 min (mean ± standard deviation (SD)), respectively. In contrast, although the T_1/2_ for the HV yogurt was higher than that of the CT yogurt, it was not statistically different (*p* = 0.12). There was no significant difference between the β factor calculated from the gastric emptying curve of the three yogurts.

**Figure 1:**
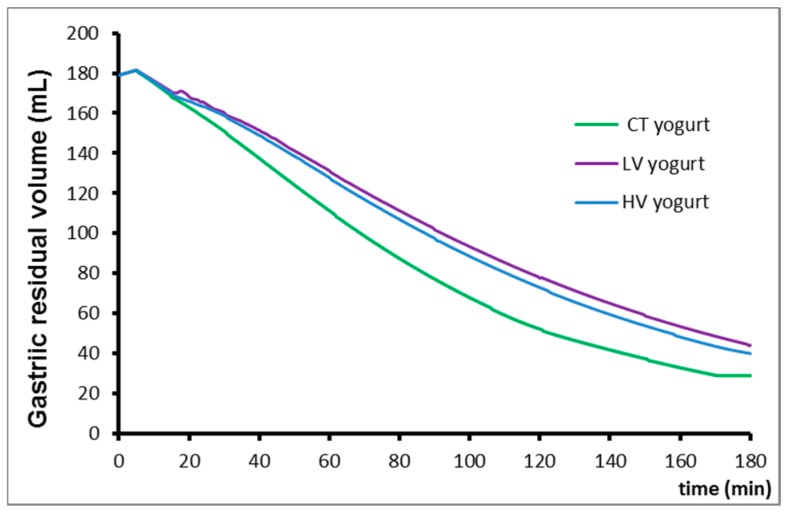
Gastric residual volume for the three yogurts. CT, LV, HV stand for control, low viscosity and high viscosity yogurts, respectively. T_1/2_ means gastric emptying half-time.

The modelling of the mean gastric empting curves is presented in Figure 1.

**Figure 2:**
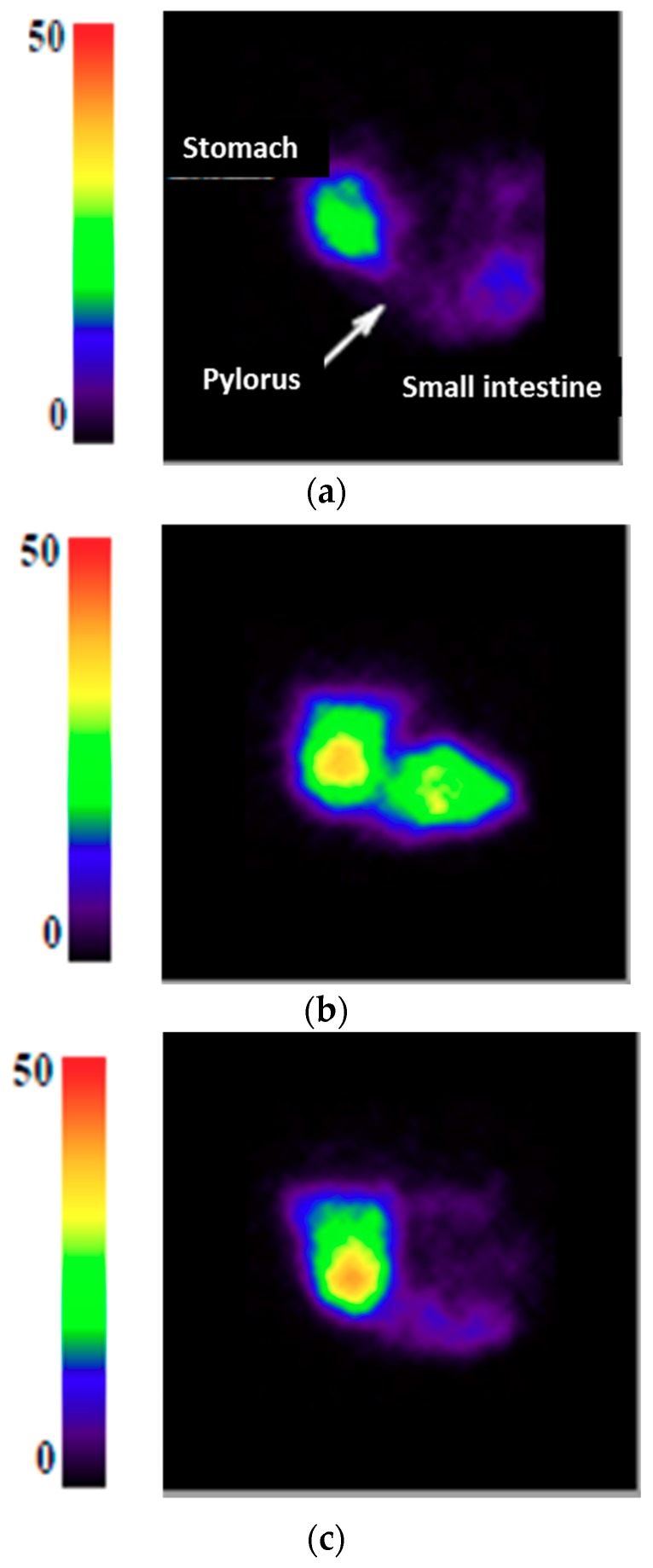
Images of the gastrointestinal region taken 60 min after meal ingestion for the control yogurt (**a**) the low viscosity yogurt; (**b**) and the high viscosity yogurt; and (**c**) The output of the radio-isotopic counter was expressed through a scale ranging from 0 to 50.

A difference of filling of the proximal part of the small intestine according to the type of yogurt was observed. The control yogurt did not stay in the duodenum but was spread all over the first segments of the small intestine. In contrast, the high viscosity yogurt accumulated in the proximal part of the small intestine, whereas the low viscosity yogurt had a similar behavior to that of the control, as shown in Figure 2. This was only an observation made for eight out of 11 pigs; quantifying this phenomenon would require a specific experiment.

### 3.2. Dynamic In Vitro Digestion

**Figure 3:**
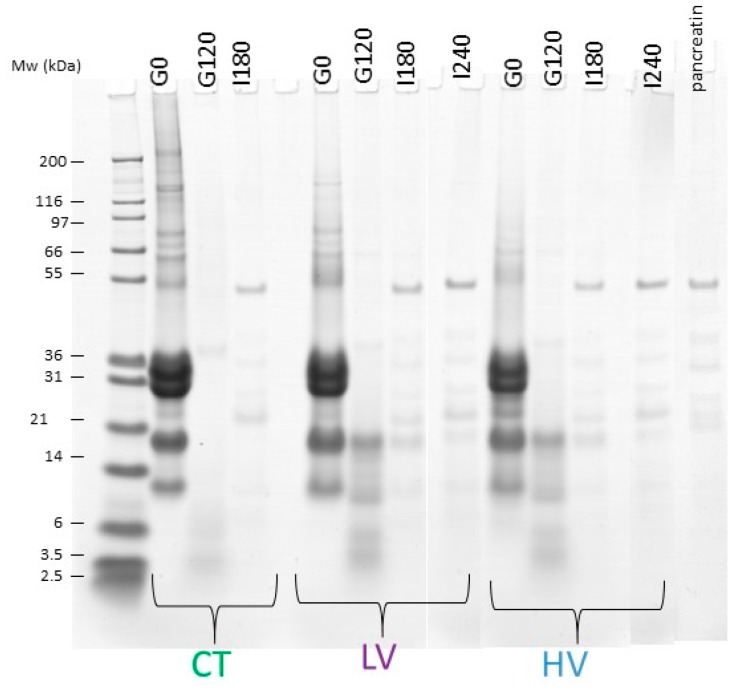
SDS-PAGE of the undigested 3 yogurts, i.e., control (CT), low (LV), and high (HV) viscosity before digestion (G0), after 120 min gastric digestion (G120) and 180 and/or 240 intestinal digestion (I180, I240).

**Figure 4:**
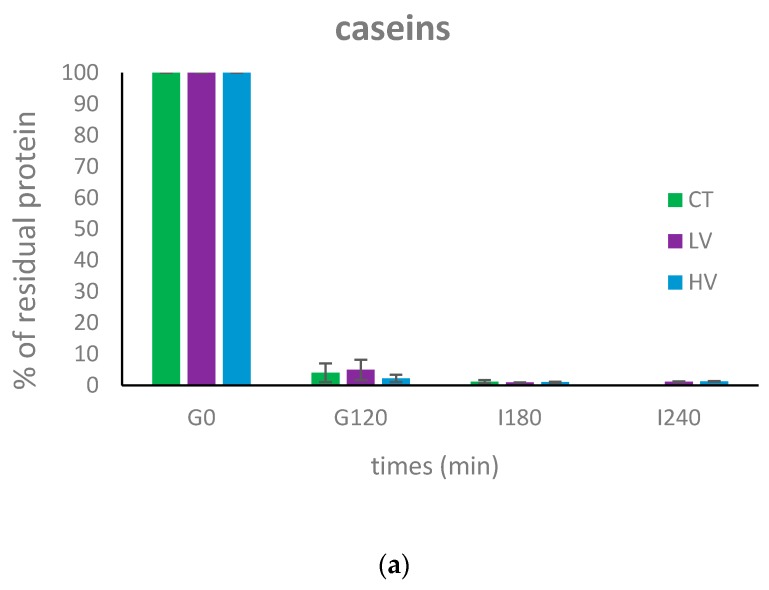

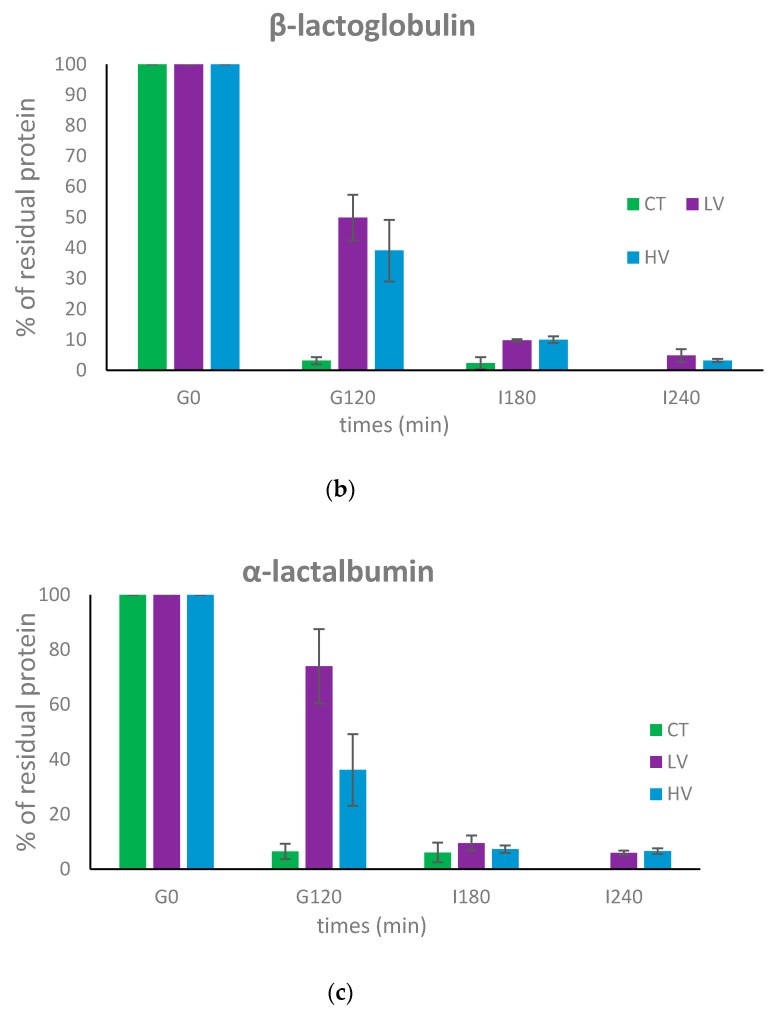
Residual % of casein (**a**), α-lactoglobulin (**b**), and α-lactalbumin (**c**) before digestion (G0), after 120 gastric digestion (G120) and after 180 (I180) and 240 (I240) min of intestinal digestion in the control (green), low viscosity (violet), and high viscosity (blue) yogurts.

Figure 3 shows the evolution of the three main milk proteins during gastrointestinal digestion. The decrease in the band intensity is due to the concomitant dilution by the secretions and proteolysis occurring during the digestion process. Caseins, shown in Figure 4a, were extensively degraded during gastric digestion and almost totally disappeared in all the samples after 120 min of gastric digestion. The percentage of residual caseins at the end of the gastric phase, compared to the amount of caseins in the undigested product, represented 4%, 4.9%, and 2.2% for the control, the low viscosity and the high viscosity yogurt, respectively. Casein digestion was finalized in the intestinal compartment resulting in the total disappearance of the caseins bands after 180 min of gastrointestinal digestion.

The situation was different for β-lactoglobulin, where whey was the major protein (Figure 4b). In comparison, β-lactoglobulin was extensively hydrolyzed in the control yogurt with only 3.1% remaining after 120 min of gastric digestion, this protein was more resistant to digestion for the low viscosity and high viscosity with 49.8% and 39.1% remaining at 120 min of the gastric phase, respectively. After 180 min (CT) and 240 min (LV and HV) of intestinal digestion, β-lactoglobulin was shown to be extensively hydrolyzed in the three yogurts with 2.3%, 4.8%, and 3.2% remaining for the control, the low viscosity, and the high viscosity yogurt, respectively.

Finally, α-lactalbumin was sensitive to gastric digestion in the control yogurt with 6.4% being intact after 120 min digestion (Figure 4c). It was shown to be more resistant during gastric digestion in the low viscosity yogurt (73.9%), whereas it was partly digested in the high viscosity yogurt (36.2%). After 180 min (CT) and 240 min (LV and HV) of intestinal digestion, α-lactalbumin was also shown, like β-lactoglobulin, to be highly degraded in the three different yogurts with 6%, 6%, and 6.5% remaining for the control, the low viscosity, and the high viscosity yogurt, respectively.

## 4. Discussion

The present study indicated that enriching a yogurt with protein and fiber slows down gastric emptying. Indeed, the control yogurt, that has an identical caloric content as the low viscosity yogurt but a 2.5-fold reduction in protein content and no fiber, exhibited a 20% lower gastric emptying half-time (57.7 min vs. 72.7 min). This confirms the role that enrichment with milk proteins and fiber plays on the transit of the food in the gastrointestinal tract. Gastric emptying half-time of the control yogurt was lower than that of the high viscosity yogurt, but the difference was not statistically significant. Low and high viscosity yogurts did not show statistical differences in gastric emptying indicating that viscosity of the undigested yogurt was not a crucial parameter impacting gastric emptying in the present study. Finally, no significant differences were observed between the β factors calculated from the gastric emptying curve of the three yogurts. The β factor reflects the shape of the emptying curve and can be highly impacted by the physical state of the food (e.g., liquid vs. solid). In the present case, the three test foods were all gels and exhibited emptying curves of exponential power nature, typical for this kind of material. It should also be noted that a high inter-individual variability was observed in this in vivo assay, probably limiting the significance of the differences observed between the three yogurts. Therefore, a new experiment with a higher number of pigs would be required to emphasize the differences of behavior of the three yogurts in the gastrointestinal tract and see whether the high viscosity one is different than that of the control. In the hierarchy of satiating effects, proteins have been shown to be more effective than carbohydrates and fat [36]. The possible physiological mechanisms underlying this effect include diet induced thermogenesis [37] and gastrointestinal hormonal signaling [38], although more recent studies have suggested that the sensory experience of ingesting protein is also important [26,39].

The limit of the present study was that low and high viscosity yogurts differed from the control not only by their protein content but also by the presence of fibers. Hence, it is impossible to conclude which of these two components had the most important effect on gastric emptying. The effect of fibers on gastric emptying highly depends on the nature and the characteristics of the molecule and the interaction it can have with the other food constituents. A review systematically investigated the available literature on the relationship between dietary fiber types, appetite, acute and long-term energy intake, and body weight [16]. It was observed that more viscous fibers presumably affect subjective appetite and acute energy intake, whereas no evident association between physicochemical properties and long-term energy intake or body weight was found. A recent systematic review on the effect of fiber on satiety showed that most of the acute fiber treatment (78%) did not reduce food intake [14]. A recent study on the combined effect of high protein content (casein or pea) and dietary fiber (pectin) on food intake was carried out on obese rats [40]. It showed that dietary pectin, but not high protein, decreased food intake and decreased body weight. However, the protein content was two times lower and the fiber content and four times higher than in the present study. Therefore, further research on decoupling the two parameters is mandatory to generate a definite conclusion.

Interestingly, the low and high viscosity yogurts had different behaviors when entering in the small intestine, even though they exhibited similar gastric emptying half-times. The high viscosity yogurt accumulated in the proximal part of the small intestine, whereas the low viscosity yogurt did not stay in the duodenum but spread along the first segments of the small intestine. This could result in different intestinal transit time of the two yogurts. Unfortunately, although this phenomenon was visible for eight out of the 11 pigs used in the study, in the absence of a reference anatomical imaging co-registered with gamma-scintigraphy, we were not able to quantitatively assess the intestinal time of transit of the different yogurts and could not be simulated in the in vitro dynamic digestions. Nevertheless, our data strongly suggest that the effect of yogurt viscosity is not on the time of residence of the yogurt in the stomach, but further in the small intestine. To our knowledge, such a result has never been published.

Dynamic in vitro digestion using the T_1/2_ and β parameters, determined in vivo, allowed for the following of the evolution of the three main milk proteins i.e., casein, β-lactoglobulin, and α-lactalbumin in the gastrointestinal tract. Caseins were shown to be extensively hydrolyzed in the stomach, compared to whey proteins as previously described [35,41]. Caseins have a flexible and loose structure that makes them highly sensitive to digestive enzymes [42]. In contrast, the globular structure of whey proteins make them partly resistant to digestion by pepsin [43,44]. In the present case, the heat treatment applied to milk during yogurt manufacture made these proteins less resistant to digestion than the native form, due to conformational changes as previously shown [45,46,47]. In vitro digestion also showed some differences in the hydrolysis of whey proteins between the control yogurt and the low or high viscosity yogurt. Indeed β-lactoglobulin was less affected by pepsin in the gastric phase for the low and high viscosity yogurts than for the control yogurt where the protein was extensively hydrolyzed. This difference might be explained by the differences of enzyme/substrate ratio that were present between the control yogurt and the low or high viscosity yogurts. The control yogurt had 2.5 times less proteins than the two other yogurts, and for the three yogurts, the amount of pepsin provided during digestion was the same. Interestingly, whey proteins appeared to be more sensitive to pepsin hydrolysis when present in the high viscosity versus the low viscosity yogurt. This might be explained by the different behaviors of the two yogurts observed by gamma scintigraphy when entering the small intestine. Thus, it is possible that gastric conditions affect the microstructure of the two gels, leading to differences in the accessibility of whey proteins in the chyme.

Finally, dynamic in vitro digestion also demonstrated that, even though the amounts of milk proteins were 2.5 higher in the low or high viscosity yogurt than in the control one, the milk protein enriched yogurts are still well digested. This confirms that dairy proteins, even at high concentration, are highly digestible, as has been shown previously [48]. Indeed, most of the available studies indicated digestibility of milk proteins to be around 95% [49] (which means from 5% of undigested proteins at the extremity of the small intestine), which is coherent with the values we have found in the present study. Together with excellent profiles in essential amino acids, it emphasizes that milk proteins are very high in nutritional properties.

In the present study, we have not investigated the hormonal profile after the ingestion of the experimental diets. It could be highly interesting to repeat the experiment to identify the alteration in GLP-1, Cholecystokinin (CCK), ghrelin, etc., and other peptides in relation to emptying. This could clarify the mechanisms of action of the milk protein enriched yogurts in generating satiety.

## Figures and Tables

**Table 1:** Composition and texture of the three yogurts.

	Control	Low Viscosity	High Viscosity
Protein (g/100 g)	3.1	8.1	8.1
Lipid (g/100 g)	0.1	0.2	0.2
Sugar (g/100 g)	17.3	11.6	11.6
Fiber (g/100 g)	0	2.5	2.5
Starch (%)	0.53	0.18	0.18
Energy (kcal/100 g)	82	85	85
Texture	standard	liquid	thick liquid

**Table 2:** Apparent viscosity of the three yogurts measured 4 days (D + 4) and 15 days (D + 15) after manufacture.

Viscosity_app_ (Pa∙s)	CT	LV	HV
D + 4	1.26 ± 0.12	0.32 ± 0.04	2.20 ± 0.11
D + 15	1.30 ± 0.05	0.37 ± 0.04	2.07 ± 0.20

Note: Viscosity_app_, apparent viscosities; CT, control yogurt; LV: low viscosity; HV: high viscosity.

**Table 3:** Gastro-intestinal parameters for in vitro dynamic digestions.

**Gastric Conditions (37 °C)**			
Simulated Gastric Fluid (SGF) (stock solution adjusted at pH 6.5)	Na+		100 mmol/L
	Ca^2+^		1 mmol/L
Fasted state/initial conditions	SGF		24 mL
	pH		1.8
Yogurt	Ingested amount		150 g
Gastric pH (acidification curve)	pH = 1.68 + 3.82(−t/42) (with t: time after ingestion in min)		
SGF + pepsin (porcine)	Pepsin		2000 U/mL of gastric content
	Flow rate		1 mL/min from 0 to 5 min
	Flow rate		0.5 mL/min from 5 to 180 min
Gastric emptying (Elashoff fitting)	CT	T_1/2_ *	58 min
		β	1.1
	LV	T_1/2_	73 min
		β	1.0
	HV	T_1/2_	65 min
		β	1.1
**Intestinal Conditions (37 °C)**			
Simulated Intestinal Fluid (SIF) (stock solution adjusted at pH 6.2)	Na^+^		100 mmol/L
	Ca^2+^		1 mmol/L
Intestinal pH	pH		6.6
SIF + bile (bovine)	Bile		4% from 0 to 30 min
	Bile		2% from 30 min to the end
	Flow rate		0.5 mL/min from 0 to the end
SIF + pancreatin (porcine)	Pancreatin		7%
	Flow rate		0.25 mL/min from 0 to the end
Intestinal emptying (Elashoff fitting)	T_1/2_		160 min
	β		1.6

* T_1/2_ = gastric emptying half-time.

**Table 4:** Gastric emptying half-time (T_1/2_) and shape of the emptying curve (β) observed on the 3 yogurts.

	T_1/2_ (min)	β
Control	57.7 ± 3.9	1.1 ± 0.05
Low viscosity	72.7 ± 5.1 *	1.0 ± 0.04
High viscosity	65.3 ± 3.5	1.1 ± 0.03

* indicates a statistically significant difference (*p* < 0.05) between the control and the low viscosity yogurt.

## Supplementary Materials

The following are available online at http://www.mdpi.com/2072-6643/10/9/1308/s1, Video S1: Yogurt gastric emptying followed by gamma-scintigraphy.
